# Supplementary material for: Mental health burden among females living with HIV and AIDS in sub-Saharan Africa: A systematic review
Source: PLOS Glob Public Health. 2024 Feb 1;4(2):e0002767. doi: 10.1371/journal.pgph.0002767 (PMC10833589; doi:10.1371/journal.pgph.0002767)
Supplement: S1 Data — (DOCX) [file pgph.0002767.s002.docx]

RESPECTIVE DATABASES, SEARCH TERMS, AND SEARCH HISTORIES FOR SYSTEMATIC REVIEW

| Electronic Databases | Search Strategy/Terms |
| --- | --- |
| PubMed | ((("Mental Health"[Mesh] OR "Mental health"[tw] OR "Mental wellbeing"[tw] OR "Psychological wellbeing"[tw] OR Depress*[tw] OR Anxiety[tw] OR "Mental disorder*"[tw] OR "Psychological disorder*"[tw] OR "Mental illness*"[tw] OR "Psychological distress"[tw] OR "Mental distress"[tw] OR "Emotional distress"[tw]) AND ("Female"[Mesh] OR Female*[tw] OR Woman [tw] OR Women [tw]))  AND ("HIV"[Mesh] OR HIV/AIDS [tw] OR "HIV positive" [tw] OR "HIV and AIDS" [tw] OR  "Acquired immunodeficiency syndrome" [tw] OR "Human immunodeficiency virus")) AND  ("Africa"[Mesh] OR Africa [tw] OR "Sub Saharan Africa" [tw]) |
| Scopus | (TITLE-ABS-KEY ("Mental health" OR "Mental wellbeing" OR "Psychological wellbeing" OR Depress* OR Anxiety OR "Mental disorder*" OR "Psychological disorder*" OR "Mental illness*"OR "Psychological distress" OR "Mental distress" OR "Emotional distress") AND TITLE-ABSKEY(Female* OR Woman OR Women) AND TITLE-ABS-KEY(HIV/AIDS OR "HIV Positive" OR "HIV and AIDS" OR "Acquired immunodeficiency syndrome" OR "Human immunodeficiency virus") AND TITLE-ABS-KEY(Africa OR "Sub Saharan Africa")) |
| MEDLINE with full text | ( "Mental health" OR "Mental wellbeing" OR "Psychological wellbeing" OR Depress* OR Anxiety OR "Mental disorder*" OR "Psychological disorder*" OR "Mental illness*"OR "Psychological distress" OR "Mental distress" OR "Emotional distress" ) AND ( Female* OR Woman OR Women ) AND ( HIV/AIDS OR “HIV Positive” OR “HIV and AIDS” OR “Acquired immunodeficiency syndrome” OR “Human immunodeficiency virus” ) AND ( Africa OR “Sub Saharan Africa” ) |
| Academic search complete | ( "Mental health" OR "Mental wellbeing" OR "Psychological wellbeing" OR Depress* OR Anxiety OR "Mental disorder*" OR "Psychological disorder*" OR "Mental illness*"OR "Psychological distress" OR "Mental distress" OR "Emotional distress" ) AND ( Female* OR Woman OR Women ) AND ( HIV/AIDS OR “HIV Positive” OR “HIV and AIDS” OR “Acquired immunodeficiency syndrome” OR “Human immunodeficiency virus” ) AND ( Africa OR “Sub Saharan Africa” ) |
| Health Source: Nursing Academic education | ( "Mental health" OR "Mental wellbeing" OR "Psychological wellbeing" OR Depress* OR Anxiety OR "Mental disorder*" OR "Psychological disorder*" OR "Mental illness*"OR "Psychological distress" OR "Mental distress" OR "Emotional distress" ) AND ( Female* OR Woman OR Women ) AND ( HIV/AIDS OR “HIV Positive” OR “HIV and AIDS” OR “Acquired immunodeficiency syndrome” OR “Human immunodeficiency virus” ) AND ( Africa OR “Sub Saharan Africa” ) |
