# Supplementary material for: Mental health burden among females living with HIV and AIDS in sub-Saharan Africa: A systematic review
Source: PLOS Glob Public Health. 2024 Feb 1;4(2):e0002767. doi: 10.1371/journal.pgph.0002767 (PMC10833589; doi:10.1371/journal.pgph.0002767)
Supplement: Attachment — Submitted filename: PGPH-D-23-01441_reviewer.pdf [file pgph.0002767.s003.pdf]

PLOS Global Public Health

Mental Health Burden among Females Living with HIV and AIDS in sub-Saharan Africa: A systematic review.

--Manuscript Draft--

**Manuscript Number:**	PGPH-D-23-01441
**Article Type:**	Research Article
**Full Title:**	Mental Health Burden among Females Living with HIV and AIDS in sub-Saharan Africa: A systematic review.
**Short Title:**	Mental Health Burden among Females Living with HIV/AIDS
**Corresponding Author:**	Dorothy Serwaa Boakye, PGDE,MA,Mphil, RN<br>University of Education Winneba Faculty of Science Education<br>Winneba, Central GHANA
**Order of Authors:**	Dorothy Serwaa Boakye, PGDE,MA,Mphil, RN
	Mawuko Setordzi, MPhil, RN
	Gladys Dzansi, PhD, MPhil, RN
	Samuel Adjorlolo, PhD, MPhil, RN
**Keywords:**	Mental health burden; Depression; Anxiety; Females; HIV/AIDS; Africa; Systematic review
**Abstract:**	Background Mental health problems, particularly depression and anxiety are common in women and young girls living with HIV and AIDS particularly in low- and middle-income (LMICs) countries where women’s vulnerability to psychiatric symptoms is heightened due to the prevalent intersectional stressors such as stigma and intimate partner violence. However, no synthesized evidence exists on the mental health burden of females living with HIV and AIDS (FLWHA) in Africa. Objective This systematic review aimed to synthesize the current evidence on the mental health burden among females living with HIV/AIDS in sub-Saharan Africa. Method A systematic literature review of articles published from 2013–2023 was conducted using the Preferred Reporting Items for Systematic Reviews and Meta-Analyses guidelines (PRISMA). Five electronic databases; PubMed, MEDLINE with full text, Scopus, Academic search complete, and Health Source: Nursing academic education were searched for articles published in English. An adapted Joanna Briggs Institute (JBI) etiology review methodology was followed in conducting this review. Findings Nineteen articles (15 quantitative, 3 qualitative, and 1 case study) from over 7 countries met the inclusion criteria. The majority of the studies' quality was determined to be moderate using the JBI critical evaluation tool for studies reporting prevalence data, qualitative studies, and case studies. The pooled prevalence of depression ranged from 5.9 to 61% and anxiety 28.9 to 61%. Mental health burden was a logical outcome of HIV diagnosis. Correlates and predictors of mental health outcomes in the context of HIV/AIDS were identified as Intimate partner violence (IPV), Stigma, childhood traumas, sexual abuse, poverty, unemployment, and social isolation. Social support and resilience were identified as protective factors against mental illness in FLWHA. Mental illness negatively impacted virologic outcomes of FLWHA, resulted in delayed initiation of antiretroviral therapy treatment and increased mortality but had no impact on immune reconstitution (CD4 count) in the face of ART adherence. Conclusion/ Recommendation Given the high prevalence rates of depression and anxiety and their relationship with HIV progression, it is crucial that mental health care services are integrated into routine HIV care. Mental health care should highlight interventions that address the unique cultural needs of FLWHA in Africa.
**Additional Information:**	
**Question**	**Response**
**Financial Disclosure**	The author(s) received no specific funding for this work

Powered by Editorial Manager® and ProduXion Manager® from Aries Systems Corporation

Enter a financial disclosure statement that describes the sources of funding for the work included in this submission and the role the funder(s) played. This includes grants and any commercial funding of the work or authors.<br><br>This statement will be typeset if the manuscript is accepted for publication.<br><br>*Please review the submission guidelines and the instructions link below for detailed requirements and guidance.*	
**Competing Interests**<br><br>On behalf of all authors, disclose any competing interests that could be perceived to bias this work.<br><br>This statement will be typeset if the manuscript is accepted for publication.<br><br>*Please review the instructions link below and PLOS Global Public Health's competing interests policy to determine what information must be disclosed at submission.*	The authors have declared that no competing interests exist.
**Data Availability**<br><br>Before publication, Authors are required to make fully available and without restriction all data underlying their findings. Please see our PLOS Data Policy page for detailed information on this policy.<br><br>A **Data Availability Statement**, detailing where the data can be accessed, is required at first submission. Insert your Data Availability Statement in the box below.	All data underlying the findings in this manuscript has been submitted as supporting information.

Powered by Editorial Manager® and ProduXion Manager® from Aries Systems Corporation

Please see the data reporting section of our submission guidelines for instructions on what you need to include in your Data Availability Statement.<br><br>This statement will be typeset if the manuscript is accepted for publication.<br><br>*PLOS allows rare exemptions to address legal and ethical concerns. If you have legal or ethical restrictions, please use the box below to detail these in full sentences for the Journal team to consider.*	

Powered by Editorial Manager® and ProduXion Manager® from Aries Systems Corporation

Manuscript Click here to access/download;Manuscript;Manuscript (Mental Health Burden in FLWHA).docx

Mental Health Burden among Females Living with HIV and AIDS in sub-Saharan Africa: A systematic review.

Dorothy Serwaa Boakye[1,3] Mawuko Setordzi[2,3] Gladys Dzansi[3], Samuel Adjorlolo [3,4]

1. Department of Health Administration and Education, University of Education, Winneba, Ghana
2. Department of Nursing, Presbyterian Nursing, and Midwifery Training College, Box 47, Dormaa Ahenkro -Bono Region, Ghana
3. School of Nursing and Midwifery, College of Health Sciences, University of Ghana, Legon.
4. Research and Grant Institute of Ghana, Legon, Ghana.

Corresponding authors
Name: Dorothy Serwaa Boakye and Mawuko Setordzi

Email: dsboakye@uew.edu.gh and mwksetordzi3@gmail.com

Abstract

Background- Mental health problems, particularly depression and anxiety are common in women and young girls living with HIV and AIDS particularly in low- and middle-income (LMICs) countries where women's vulnerability to psychiatric symptoms is heightened due to the prevalent intersectional stressors such as stigma and intimate partner violence. However, no synthesized evidence exists on the mental health burden of females living with HIV and AIDS (FLWHA) in Africa.

Objective-This systematic review aimed to synthesize the current evidence on the mental health burden among females living with HIV/AIDS in sub-Saharan Africa.

Method- A systematic literature review of articles published from 2013–2023 was conducted using the Preferred Reporting Items for Systematic Reviews and Meta-Analyses guidelines (PRISMA). Five electronic databases; PubMed, MEDLINE with full text, Scopus, Academic search complete, and Health Source: Nursing academic education were searched for articles published in English. An adopted Joanna Briggs Institute (JBI) etiology review methodology was followed in conducting this review.

Findings- Nineteen articles (15 quantitative, 3 qualitative, and 1 case study) from over 7 coun[illegible]s met the inclusion criteria. The majority of the studies' quality was determined to be moderate using the JBI critical evaluation tool for studies reporting prevalence data, qualitative studies, and case studies. The pooled prevalence of depression ranged from 5.9 to 61% and anxiety 28.9 to 61%. Mental health burden was a logical outcome of HIV diagnosis. Correlates and predictors of mental health outcomes in the context of HIV/AIDS were identified as Intimate partner violence (IPV), Stigma, childhood traumas, sexual abuse, poverty, unemployment, and social isolation. Social

support and resilience were identified as protective factors against mental illness in FLWHA. Mental illness negatively impacted virologic outcomes of FLWHA, resulted in delayed initiation of antiretroviral therapy treatment and increased mortality but had no impact on immune reconstitution (CD4 count) in the face of ART adherence.

Conclusion/ Recommendation-Given the high prevalence rates of depression and anxiety and their relationship with HIV progression, it is crucial that mental health care services are integrated into routine HIV care. Mental health care should highlight interventions that address the unique cultural needs of FLWHA in Africa.

KEYWORDS: Mental health burden, Depression, Anxiety, Females, HIV/AIDS, Africa

INTRODUCTION

Since the beginning of the HIV/AIDS epidemic, women and girls have been most infected and affected. The joint United Nations Program on HIV/AIDS reports show that globally, more than half (20.2 million) of the 37.7 million people living with HIV and AIDS are females [1]. The numbers recorded in sub-Saharan Africa are even more devastating. Approximately, two-thirds of the estimated 25.6 million people currently living with HIV and AIDS in sub-Saharan Africa are females [1,2].

Mental health conditions affect females living with HIV and AIDS (FLWHA) at substantially higher rates compared with men living with HIV and AIDS (MLWHA) and HIV-negative women [3–6]. Thus, the established gender disparity in mental health disorders such as depression and anxiety is disproportionately increased in the context of HIV and AIDS [7]. Given the psychological distress associated with managing and coping with the illness, an HIV diagnosis may lead to mental illness symptomatology or even exacerbate pre-existing mental illness symptoms in women [4,8].

FLWHA’s relative vulnerability to mental health conditions may be due to several intersectional stressors such as unemployment, financial stressors, decreased psychological resilience, limited social support, and increased susceptibility to neuropsychiatric side effects of some antiretroviral therapies such as zidovudine and abacavir [9–11]. Additionally, FLWHA in low- and middle-income countries have higher rates of intimate partner violence, stigma, discrimination, and social prejudice [10,12,13] as well as increased psychosocial stress with disclosure of HIV status and management of sexual and reproductive health concerns [13,14].

Higher rates of depressive and anxiety symptoms have been reported amongst FLWHA in several studies [3–7]. For instance, 82% of survey respondents in a study of 832 FLWHA from 94

countries reported having symptoms of depression, and participants indicated that their mental health concerns rose 3.5-fold after receiving an HIV diagnosis [15]. Furthermore, 28.9% of 357 FLWHA ages 18 years and above in an Ethiopian study experienced moderate to high levels of anxiety, as measured by a score of at least 11 on the Hospital Anxiety and Depression Scale (HADS-A) [6]. As depressive and anxiety disorders are frequently underdiagnosed among persons living with HIV and AIDS (PLWHA), these rates are likely to be higher than reported [16].

It must be noted that the highest burden of HIV-related mental illness is in Afr[illegible] [17]. However, most of the mental health interventions for this population have been developed and evaluated in resource-rich settings and with a limited focus on FLWHA. Along with this limitation even exist a major gap as there is a lack of systematic reviews that synthesize the available research evidence on mental health illness among FLWHA in Africa to direct policymakers to implement cost-effective and evidenced-based gender-specific interventions that are peculiar to our cultural context as Africans. Our objective was to synthesize the current understanding of the specific mental health problems experienced by FLWHA in Africa. Our study may have implications for the United Nations 95-95-95 treatment target by 2025 since the evidence shows FLWHA with untreated mental illness are less likely to achieve good antiretroviral therapy (ART) adherence, and more likely to develop virologic failure and HIV progression [18].

1.1. Aim of the review

The main aim of this review was to synthesize the current understanding of the specific mental health problems among FLWHA in Africa

1.2. Objectives of the review

1. Determine the prevalence of depression and anxiety among FLWHA.
2. Describe the impact of HIV/AIDS on the mental health burden of FLWHA.
3. Identify the risks, and protective factors of mental health problems among FLWHA
4. Describe the impact of mental health burden on HIV progression among FLWHA

1.3. Review question

The main systematic review question which was asked to guide the review methodology was: “Does HIV AND AIDS status impact negatively on the mental health of FLWHA?”

Table 1. Review question in PEO format

PEO format	Concepts
**P-**Population-	Females
**E-**Exposure -	HIV/AIDS
**O-**Outcome-	Mental Health burden

Table 2. Search strategy for PubMed using Boolean Operators (OR & AND)

PEO Format	Combined Text words and MeSH Words
**P- population**	"Female"[Mesh] OR Female*[tw] OR Woman [tw] OR Women [tw]
**E- exposure**	"HIV"[Mesh] OR HIV/AIDS [tw] OR “HIV-positive” [tw] OR “HIV and AIDS” [tw] OR “Acquired immunodeficiency syndrome” [tw] OR “Human immunodeficiency virus”

**O- outcome**	"Mental Health"[Mesh] OR "Mental health"[tw] OR "Mental wellbeing"[tw] OR "Psychological wellbeing"[tw] OR Depress*[tw] OR Anxiety[tw] OR "Mental disorder*"[tw] OR "Psychological disorder*"[tw] OR "Mental illness*"[tw] OR "Psychological distress"[tw] OR "Mental distress"[tw] OR "Emotional distress"[tw]
	"Africa"[Mesh] OR Africa [tw] OR "Sub Saharan Africa" [tw]

2.0 Methods

This systematic review adheres to the Preferred Reporting Items for Systematic Reviews and Meta Analysis (PRISMA) guidelines [19]. Before the commencement of the review, we conducted an initial search on Prospero and Cochrane Library using the main concepts of the topic in **Table 2** to ascertain if the systematic review has been done already to avoid unintentional duplication and redundancy. We noticed that systematic reviews were conducted in the general population but not specific to FLWHA. Again, this search gave us a fair idea about the availability of literature on the topic. During the building process for the search terms, relevant synonyms were identified for each of the main concepts as shown in **Table 2**. All the key concepts and their synonyms were combined to construct a comprehensive search strategy for the identified databases. The study focused on studies on Mental health burden in females living with HIV/AIDS in Africa.

2.1 Inclusion and exclusion criteria

Articles were included in the review if they focused on mental health problems in females living with HIV/AIDS between 17 and 76 years and published in English in the last ten years from 2013 to April 2023. Articles focused on pregnant and post-natal women were excluded because 1)

systematic reviews exist in the literature in Africa on this population and 2) pregnancy and its associated hormonal imbalance are considered covariates to mental health burden in FLWHA [20,21]. The details of the criteria for inclusion and exclusion have been described in detail in Table 3 and 4.

Table 3

**Inclusion criteria**
1. Mental health in females between 17 and 76 years living with HIV/AIDS
2. Scholarly articles on depression and anxiety among females living with HIV/AIDS
3. Studies that employed quantitative, or qualitative research designs
4. Scholarly articles published in English
5. Scholarly articles published within the last ten years (2013-2023)
6. Articles accessible as full text

Table 4

**Exclusion criteria**
1. Studies describing mental health burden in pregnant females living with HIV/AIDS
2. Studies describing mental health burden in post-natal women living with HIV/AIDS
3. Studies specifically focused on mental health interventions among FLWHA
4. Review articles

2.2 The electronic database search

Five (5) major databases were selected for the electronic search. A systematic search of electronic databases using pre-defined search terms, supplemented by hand-searching, was undertaken in April 2023. These databases included: PubMed, Scopus, MEDLINE with full text, Academic search complete, and Health Source: Nursing academic education. Additionally, the reviewers searched Google Scholar engine, WHO website, UNAIDS website, Ghana's Mental Health Authority website and app[illegible]ed institutional websites reporting findings and statistics on HIV/AIDS and mental illness for both grey and published literature. Also, the reference lists of the papers that were chosen for inclusion were used as a snowball for additional studies. Two reviewers (DSB & MS) conducted the final electronic search independently in the selected databases on 24 April 2023 with the help of the designed search strategy.

2.3 Conducting the electronic search

First, articles retrieved from the various relevant electronic databases and the other sources were title screened by the reviewers (DSB & MS) to determine whether they were relevant for inclusion. Second, the abstracts of the respective articles were also screened to ascertain whether their findings will contribute meaningfully to the research questions of the review. Third, the full text of the respective articles was screened and those that were found significant were added. Duplicates were removed manually during the selection process and also by the use of a software called Rayyan.

2.4 Selection of Studies and Data Extraction

All titles and abstracts identified through electronic database searching and hand searching were examined for relevance. Full text articles of any potentially relevant studies were obtained and

assessed for inclusion based on the above inclusion criteria. Where uncertainty arose within the examination process, full text articles were subjected to further scrutiny by the inclusion of a third reviewer (S.A). S.A who doubled as project supervisor and reviewer, also assisted with validation and review of a subsection of identified titles and abstracts. Less than 5% discrepancies arose between the authors (DBS & MS) and the third reviewer. Data from studies that met the inclusion criteria were extracted using an extraction format adapted from a recent systematic review [22]. Data extraction was carried out by the first and second authors (DSB & MS) and, scrutinized by the fourth author (S.A). Information extracted from relevant articles included publication details such as sample size and its characteristics, country, measures used, authors and year of publication and the major finding well informed by the inclusion c[illegible]ria

Fig.1. Updated PRISMA 2020 flow diagram showing the study selection process.

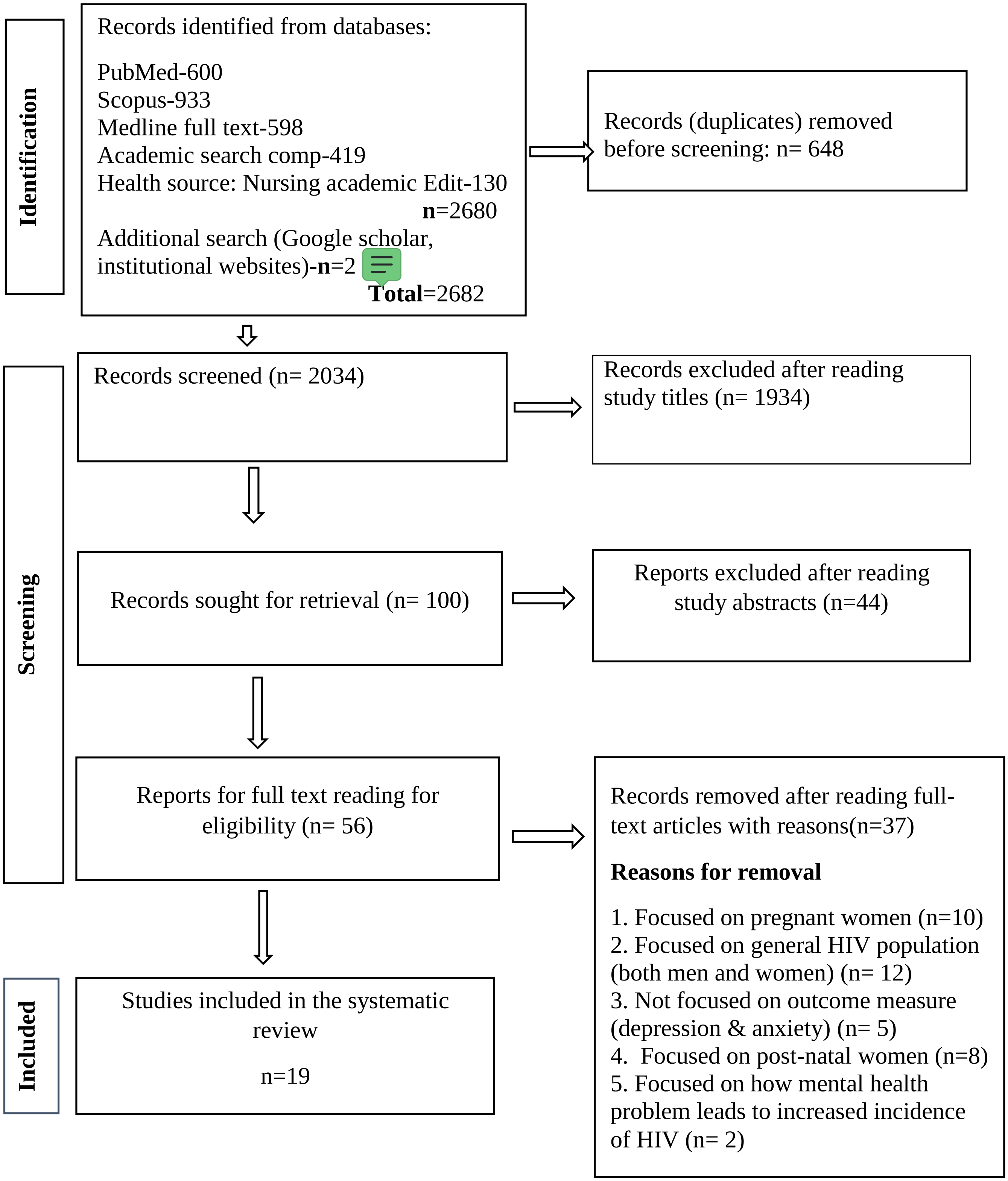

2.6. Data extraction from included studies

Using the adapted data extraction format, relevant data was retrieved from each included study for the analysis. The author and year of publication, the title of publication, the country where the study was done, the sample size and characteristics, measures used, prevalence, risks/ correlates, protective factors, impact of HIV/AIDS on mental health, and the impact of mental health on HIV progression were extracted from each including. Special attention was paid to the methods of recruitment/selection of participants, data collection approach and data analysis done for each of the studies. Data were extracted into tables with pre-specified categories as stated previously. For quantitative studies, we collected prevalence of mental health problems and its associated factors, and summarized main findings identified by the authors. For the qualitative studies, the authors collected information about the experiences of depression/anxiety, risk and coping. Data was extracted and synthesized collaboratively by two authors (MS & DSB). The fourth author (S.A) validated and made suggestions on the themes. Some were modified and subsequently included in the review **(see Table 5)**

2.7. Assessment of Quality and Risk of Bias of Included Studies

The quality of the nineteen included studies were assessed using Joanna Briggs Institute (JBI) Critical Appraisal Tools [23]. This group of tools comprised checklists adopted for a range of study designs that includes a 9-item checklist for studies reporting prevalence data [24], an 8-item checklist for case reports [25] and a 10 item checklist for qualitative studies [26]. The checklists pose a series of questions regarding presence of specific information with response options “yes = 1”, “no = 0”, “unclear = 0”, and “not applicable= not scored” to guide appraisers toward conclusions about the methodological quality of a study or analysis. **(See Table 6,7,8).**

The 9-item checklist was awarded an overall score of 9 with scores of 1-3, 4-6 and 7-9 indicating low, moderate and high quality. The 8-item checklist was awarded a score up to 8 with scores of 1-3, 4-6 and 7-8 indicating low, moderate and high quality. The overall score of the 10-item checklist was 10 with scores of 1-3, 4-6 and 7-10 indicating low, moderate and high quality. The response score of each study were summed up to determine if the study was of low, moderate or high quality.

3.0 RESULTS

3.1 Characteristics of the included studies

The search and screening processes is illustrated in Figure 1. A total of 19 articles were included in the review. All of the included studies were published between 2014 and 2023. Out of the 19 articles, 15 were quantitative, 3 qualitative and 1 case study. The participants were aged between 17 and 76 years. In all 12,299 (adolescents, adults and the aged) made up the entire sample size for the nineteen (19) studies. The major findings in the included studies have been summarized in Table 5 (summary of data extracted from included studies). Most of the studies (n = 10, 52.6%) were conducted in South Africa, followed by Kenya (n = 2, 11.1 %), Uganda (n = 2, 11.1%) whilst the remaining were conducted in Malawi (n=1,5.5%), Tanzania (n = 1, 5.5%), Ethiopia (n = 1. 5.5%) Nigeria (n = 1, 5.5%), and Parts of Sub-Saharan Africa (n = 1, 5.5%). One study was conducted both in Zambia and Uganda ([illegible] 1, 5.5%). For this review, the themes that appeared in at least two papers were kept.

3.2 Prevalence of depressive symptoms in FLWHA

Data on the prevalence of depression were published by thirteen (13) studies (68.4%) [6,27–35]. According to the data that were retrieved, the prevalence of depressive symptoms varies from 5.9 %, among HIV-positive women in Nigeria [35] to 61% among HIV-positive women in Tanzania, [34] demonstrating the lowest to the highest depression symptomatology at different study locations. In all, 688 [illegible]dolescents, adults and the aged) made up the entire sample size for the ten (10) studies. High prevalence rates were reported by two studies conducted in South Africa 56.3% [28] and Malaw[illegible].0% [29]. Relatively lower rates of depression were reported in two studies. Thus 5[illegible] in Nigeria [35] and 6.5% in South Africa [33]. In a case study conducted in Kenya on a 42 year old unmarried FLWHA, she indicated fifteen (15) symptoms corresponding to mild depression on the Beck Depression Index [36].

3.3 Prevalence of anxiety symptoms in FLWHA

The prevalence of anxiety symptomatology was reported in three (3) studies [6,29,37] representing 15.8% .The anxiety prevalence ranges from 28.9% among FLWHA in Ethiopia [6] to 61% among FLWHA in Uganda [37]. The reported anxiety prevalence in these three studies in terms of severity ranges from 61.0 % [37] in Uganda, 53.0% [29] in Malawi and 28.9%, [6] in Ethiopia. In all, 704 FLWHA made up the entire sample size for the three (3) studies.

3.4 Mostly used Mental Health Assessment Measures

Among the 19 studies that formally assessed anxiety and depression, 16 (84.2%) used standardized scales/measures while 3 (15.8%) used semi- structured interview guide [38–40]. Among the sixteen (16) studies, five (5) used the Center for Epidemiologic Studies Depression Scale (CES-D) [28,33,40–42], four used the Hopkins Symptom Checklist (HSCL-25) [27,31,34,37], three used the Patient Health Questionnaire-9 (PHQ-9) [32,43], Five studies used different scales thus,

the Patient Health Questionnaire-2 (PHQ-2) [30], Beck's Depression Inventory II (BDI-11) [35], Beck's Depression index [36], the Hospital Anxiety and Depression Scale (HADS) [6], and the Emotional distress scale [29]. Most of the scales used were developed and validated in high income settings and therefore lacked contextual (cultural and non -specificity to the HIV population) validity. The reliability coefficient of the scales was not reported in a majority of the studies.

3.5 Risks of mental illness in FLWHA

Among the 19 studies included, seventeen (17) studies (89.4 %) [6,26–34,35–39,41,43] provided data on the risks, and predictors of anxiety and depression among FLWHA in different countries. Regardless of the country, and the type of mental health outcome, these risks factors appeared corroboratory. These risk factors include higher HIV viral load (VL) category, HIV/AIDS related stigma, sexual abuse, physical intimate partner violence (IPV), childhood trauma and posttraumatic stress disorder. Other risk factors included loss of self-esteem, experiences of discrimination, loss of life partners and or other family members, post-traumatic stress disorder, childhood traumas, poverty, educational status, victimization, living with people other than direct family members, homelessness, being single or divorced.

In 11 of these studies [6,27,29–34,42–44], a univariate, multivariate or logistic regression analysis showed that sexual abuse, physical intimate partner violence, loneliness, enacted stigma, childhood trauma, post-traumatic stress disorder, young age, high viral load, having no formal education, divorce, unemployment and low social support increased the odds of depression and anxiety in FLWHA and was statistically significant ($P \leq 0.005$). However, one study [37] showed there was no significant correlation ($P \geq 0.005$) between depression/anxiety symptoms and educational status, employment status, and ART treatment status.

3.6 Protective factors of mental health problems in WLWHA

Among the 19 studies included in this review, seven (7) representing 36.8% reported on protective factors against anxiety and depression symptomatology in different countries [6,28,33,35,37,39,44]. Specifically, five (26.3%) of these studies [6,28,35,37,44] reported on social support as a buffer against anxiety and depression. However, a study conducted by Spies & Seedat [33] in South Africa among FLWHA reported that whiles childhood trauma and post-traumatic stress symptoms contributed to depression severity, resilience reduced the severity of depression, acting as a protective factor. One qualitative study found four coping mechanisms used by the participants as protective factors [39]. These FLWHA drew on indigenous local resources in their psychological re-framing of negative situations, mobilization of emotional and financial support from inter-personal networks, churches and HIV support groups. They also less commonly sought expert advice from traditional healers, medical services and or social workers.

3.7 Impact of HIV/AIDs on Mental health of FLWH

Data on the impact of HIV/AIDS on mental health outcomes of FLWHA was gathered from seven (n=7) studies representing 38.9 % [6,27,28,35,38,40,42] from four countries with relatively different sample characteristics. Among these studies, HIV/AIDS was projected as a significant correlate for depression and anxiety. For example, the study conducted by [42] observed higher rates of depressive symptomatology among women infected with HIV compared to uninfected women. However, three (n=3) studies, representing 15.8 % [32,39,41] reported that HIV/AIDS was not an independent driver of anxiety and depression among FLWHA. Two studies also reported that knowledge of HIV/AIDS-positive status and self-disclosure of HIV status was significantly associated with a decrease in depressive symptoms [41,43].

3.8 Impact of mental health on HIV progression

From the included studies for this review, 5 studies [27,34,41,43,44] reported on the impact of mental health burden on HIV progression among FLWHA. In one study conducted in Uganda and Zambia, it was noticed that depression at ART initiation was associated with an increased risk of deaths and incidence of severe anaemia among FLWHA. Again, in the same study, it was found that depression was not significantly associated with trajectory of CD4 T-cell reconstitution or the risk of immunologic failure but women with depression had increased risk of oral thrush (HR: 1.15; 0.84-1.58; p=0.38) and wasting (HR: 1.22; 95% CI: 0.81-1.83; p= 0.34), but results were not statistically significant [34]. In three studies [27,41,44], higher depression scores were associated with reduced likelihood of being virally suppressed. In Govender et al [41], this observation was more likely among younger women (AOR = 0.87, 95% CI [0.78, 0.97], $p < 0.05$) than older women. Higher score of depression was also highly significantly associated with less utilization of HIV care services and delayed initiation of ART treatment ($p < 0.01$) [41]

4.0 Discussion

Over the past few decades, there have been an increasing focus on the mental health of women and young girls living with HIV/AIDs particularly in Africa due to their heightened vulnerability to mental illness in the context of HIV [6]. Secondly, the overburdened social roles, cultural, religious and political marginalization of women [4] even in a postmodern philosophical era further threaten the mental health of these vulnerable population. It is not surprising that research and publication on the mental health of FLWHA has gained a momentum boost. Nonetheless, these research and publications are stacked with individual journals and electronic databases making it difficult for policymakers and relevant stakeholders in the field to have a comprehensive

synthesized understanding of the recent developments and evidence regarding mental health burden in FLWHA in Africa.

4.1 Prevalence of depression and anxiety.

Based on the overall pooled prevalence estimates, the prevalence of depression and anxiety was 5.9% to 61.0% and 28.9% to 61.0% respectively [6,27,28,30–34, 36,42,43, 41]

Moderately higher levels of depression (45.4%) [45] and anxiety (29.5%) [46] have been reported in the USA and Canada. This reveals the commonalities and realities of mental health issues among this population even across countries with different cultures, geopolitics and socioeconomic indices. That notwithstanding, the literature shows people who are disadvantaged socially and economically are more likely to develop depression [47]. The high prevalence rate in this review emphasizes the need to integrate mental health interventions in the HIV care cascade with special focus on women.

The pooled prevalence rates varied substantially across the individual studies due to the different measurement scales, sample size, and geographical context. Even for studies that used the same measurement scale, information on the item number, cut offs and number of included somatic symptoms varied explaining a part of the variability in the data on prevalence rates. Given the earlier concerns of under detection and under diagnosis of depressive and anxiety symptomatology in FLWHA in Africa due to cultural/religious need to suppress the condition rather than reporting and/or clinically manifesting symptoms [16,48], prevalence rates higher than what is reported in this study is possible. It is crucial that the existing measures/scales for depression and anxiety are modified and assessed for cultural validity. Dessauvagie et al., [49] has argued that scales

developed and validated in high-income settings may be inappropriate in diagnosing and quantifying the magnitude of mental health burden in resource-poor settings.

4.2 Risk factors of depression and anxiety in FLWHA.

Our study highlighted a number of intersectional stressors that were considered as the most prevalent and strong predictors of depression and anxiety in the context of HIV. These factors individually or jointly mediated at least a part of the relationship between mental health and HIV. For instance, intimate partner violence (IPV) which affects about 30% of women globally and more prevalent in sub-Saharan Africa (36% of women) [50] were identified by four studies [30–32,44] as a strong predictor of depression and anxiety in FLWHA. Stigma has shown to negatively impact the quality of life (QoL), social and psychological health and well-being of persons living with HIV [4,51]. The effect of stigma on QoL is revealed in this review as five studies identified stigma [6,29,32,34,44] to predict mental health outcomes for FLWHA in Africa. A UNAIDS [51] report uncovered that pervasive stigma and mental health issues are associated with barriers to access to and engagement in health care. Childhood trauma and posttraumatic stress disorder were implicated in two studies [33,42] to independently predict depression in FLWHA. The findings in this review align with findings from both low- and high-income countries [52–55]. Collaborative efforts of major stakeholders, health workers, and the community are needed to curb the deleterious mediating effects of these factors on the mental health of FLWHA. Community and healthcare sensitization against stigma [51], the empowerment of women and young girls [56], psychotherapeutic and cognitive behavioural interventions [4] may be crucial and advantageous at this point as such interventions have proven promising to ultimately impact the mental health outcomes of FLWHA.

4.3 Protective factors against depression and anxiety

Social support and resilience may not only have main effects on the mental health outcomes of women living with HIV/AIDS, but may moderate or buffer the negative effects of external and internal stressors on mental health of FLWHA. In one of the studies [6], Pearson's' rank correlation showed that social support had a significant negative correlation with depression. Seven studies [6,28,33–35,37,44] reported on the buffering effects of social support and resilience on depression and anxiety. In addition to their main effects, social support and resilience may function as moderators [57] to decrease the effect of stigma, intimate partner violence, sexual abuse, childhood trauma and posttraumatic stress disorders on the mental health of FLWHA. Therefore, women and young girls living with HIV in Africa may benefit greatly from mental health interventions that focus on building social support and resilience.

4.4 Impact of HIV/AIDS on the mental health of FLWHA in Africa

There is enough evidence to suggest that depression is a commonplace for FLWHA and previous research have argued that an HIV diagnosis may exacerbate preexisting mental illness or lead to depression and anxiety symptomatology given the psychosocial distress associated with managing HIV/AIDs [4,8] especially in Africa. In this current review, HIV/AIDS was projected as the consistent and enduring risk factor for depression and anxiety by seven studies. This is supported by a previous systematic review and meta-analysis [58] that mental health burden is a logical outcome of HIV/AIDs.

However, contradictory findings on HIV and mental health outcomes emerged in this review. Three studies [32,39,41] observed that HIV diagnosis was not an independent predictor of depression and anxiety. Nonetheless, its association with other risk factors are enough to evoke psychological

distress among people living with HIV/AIDS [39]. In addition, the review identified that knowledge of HIV positive status was significantly associated with a decrease in depressive symptoms [41,43].These divergent outcomes in the evidence is likely because, as discussed previously mental illness in clinical practice is highly underdiagnosed in SSA [16]. Secondly, the lack of a single validated culturally sensitive scale may also contribute to these discrepancies. Lastly, the lack of training for clinicians and the poor integration of mental health in routine HIV care contribute to the under detection and underdiagnosis of depression and anxiety [58]. Albeit, a UNAIDS [51] report also had this to say: “Depression, anxiety and HIV are mutually reinforcing”

4.5 Impact of mental health on HIV progression

The presence of depression in FLWHA in the present review was associated with delayed initiation of ART treatment, decreased likelihood of virologic suppression, increased in opportunistic infections, less utilization of HIV care services and higher HIV- related mortality [27,34,41,43,44]. Previous studies have also established a relationship between mental health and HIV disease progression [4,57]. It is possible that mental illness may have clinically measurable biological and physiological implication on viral load and physical outcomes of people living with HIV/AIDs [27]. Relationships in the opposite direction is also likely as a few studies have identified ART mediated viral suppression to be associated with a decrease in the severity of depression symptoms [59]. The findings from this review suggest that the lack of screening for and treatment of depression and anxiety among FLWHA has detrimental effects on their HIV related health outcomes. The integration of mental health services into the HIV care continuum may be an important step towards the achievement of UNAIDS 95-95-95 treatment targets by 2025 and may as well address the bidirectional links between mental health and HIV.

Strengths and weaknesses of this review

One of the strengths of this review is that it contains data from many studies, compared to earlier revi[illegible]. Also, the methodological quality of the studies included in this review is generally of moderate quality. The majority of the studies recruited collected data using standardized measuring instruments. Also, three reviewers scrutinized and worked in collaboration to select papers for inclusion and on data extraction to avoid selection or data extraction biases. Furthermore, the reviewers followed strictly, the predetermined inclusion criteria and selected only articles that met the inclusion criteria.

Regardless, this review has weaknesses and so the results should be interpreted in the light of its limitations in mind. First, due to the purposive inclusion criteria used to inform this research, the articles in the final sample cannot represent what has been done in the field of mental health and HIV. Only 5 databases were searched, and so it is possible that other relevant articles could have been left out. The exclusion of articles published in languages other than English may have contributed to evidence collection bias. However, they were eliminated because of the cost of translation.

Conclusion and recommendation

There was high prevalence of depression and anxiety among FLWHA in Africa. This review revealed that the increased burden of mental health issues among this population were mediated by intersectional stressors such as stigma, intimate partner violence and trauma. Social support and resilience appeared to moderate the relationship between HIV/AIDS, intersectional stressors and

mental health burden (depression and anxiety) among FLWHA. A mediation and moderating analysis are needed in the future to authenticate this.

There were contradictory findings on predictive main effect of HIV on mental health burden. While some studies showed an increased in depression and anxiety symptomatology in the face of an HIV diagnosis, others' findings suggested there was no significant relationship between HIV and mental health burden in FLWHA. The lack of existing true experimental findings on HIV and mental health outcomes could account for these discrepancies and the lack of conclusive evidence. Future studies should focus on establishing a causal relationship between HIV and mental illness through experimental studies.

The current evidence on depression and anxiety among FLWHA in sub-Saharan Africa is inadequate and non-widespread as the majority of the studies reporting on this public health menace is concentrated in the South and East of Africa. There also exist substantial methodological limitations in most of the studies. Small sample size, unclear justification of sample size used, and unclear methods of recruitment (sampling methods) resulting in selection bias, a decrease in the statistical power and increased alpha. Future studies should employ standardized methodological approaches, use sufficiently large samples and utilize a robust statistical analysis.

Given the frequency of stresses and mental health issues that FLWH experience, as well as the relationship between the symptoms of mental illness and suboptimal disease treatment, a cost-effective, feasible mental health interventions tailored to the unique and cultural needs of this population are crucial.

Additional information

Author Contributions: Conceptualization of study: DSB and MS. Data Extraction: DSB and MS, Validation S.A Original Draft Preparation, DSB and MS; Review and Editing: S.A and G.D

All authors have read and agreed to the published version of the manuscript.

Funding: This research received no external funding.

Institutional Review Board Statement: Not applicable.

Informed Consent Statement: Not applicable.

Data Availability Statement: All data regarding this review has been made available as a supplementary file.

Conflicts of Interest: The authors declare no conflict of interest

References

1. UNAIDS. Women and girls carry the heaviest HIV burden in sub-Saharan Africa. [Internet]. 2022. Available from: https://www.unaids.org/en/resources/presscentre/featurestories/2022/march/20220307_women-girls-carry-heaviest-hiv-burden-sub-saharan-africa.

2. WHO. World AIDS day 2022: Message of the WHO Regional Director for Africa [Internet]. 2022. Available from: https://www.afro.who.int/regional-director/speeches-messages/world-aids-day-2022

3. Opoku Agyemang S, Ninonni J, Bennin L, Agyare E, Gyimah L, Senya K, Birikorang E, Quarshie EN, Baddoo NA, Addo SA, Obiri- Yeboah D. Prevalence and associations of depression, anxiety, and stress among people living with HIV: A hospital- based analytical cross- sectional study. Health Science Reports. 2022 Sep;5(5):e754.

4. Waldron EM, Burnett-Zeigler I, Wee V, Ng YW, Koenig LJ, Pederson AB, Tomaszewski E, Miller ES. Mental health in women living with HIV: the unique and unmet needs. Journal of the International Association of Providers of AIDS Care (JIAPAC). 2021 Jan 20;20:2325958220985665.

5. Yimer S, Girma Z, Mengistu N, Shumye S. Determinants of common mental disorders among people living with hiv/aids attending art service in southern Ethiopia. HIV/AIDS - Res Palliat Care. 2021;13:917–25.

6. Yousuf A, Musa R, Isa MLM, Arifin SRM. Anxiety and Depression Among Women Living with HIV: Prevalence and Correlations. Clin Pract Epidemiol Ment Heal. 2020;16(1):59–66.

7. Gooden TE, Gardner M, Wang J, Chandan JS, Beane A, Haniffa R, et al. The risk of

mental illness in people living with HIV in the UK: a propensity score-matched cohort study. Lancet HIV. 2022;9(3):e172–81.

8. MacLean JR, Wetherall K. The Association between HIV-Stigma and Depressive Symptoms among People Living with HIV/AIDS: A Systematic Review of Studies Conducted in South Africa. J Affect Disord [Internet]. 2021;287(May 2020):125–37. Available from: https://doi.org/10.1016/j.jad.2021.03.027

9. Nakimuli- Mpungu E, Musisi S, Smith CM, Von Isenburg M, Akimana B, Shakarishvili A, Nachega JB, Mills EJ, Chibanda D, Ribeiro M, V Williams A. Mental health interventions for persons living with HIV in low- and middle- income countries: a systematic review. Journal of the International AIDS Society. 2021 Jun;24:e25722.

10. Tenkorang EY, Owusu AY, Zaami M, Langmagne S, Gyan S. Intimate partner violence and health outcomes among women living with HIV/AIDS in Ghana: a cross-sectional study. Health Education & Behavior. 2023 Jun;50(3):347-58.

11. Yimer S, Girma Z, Mengistu N, Shumye S. Determinants of Common Mental Disorders Among People Living with HIV/AIDS Attending ART Service in Southern Ethiopia. HIV/AIDS-Research and Palliative Care. 2021 Sep 16:917-25.

12. Ashaba S, Kaida A, Coleman JN, Burns BF, Dunkley E, O’Neil K, Kastner J, Sanyu N, Akatukwasa C, Bangsberg DR, Matthews LT. Psychosocial challenges facing women living with HIV during the perinatal period in rural Uganda. PloS one. 2017 May 1;12(5):e0176256.

13. Obiri-Yeboah D, Amoako-Sakyi D, Baidoo I, Adu-Oppong A, Rheinländer T. The ‘Fears’ of Disclosing HIV Status to Sexual Partners: A Mixed Methods Study in a Counseling Setting in Ghana. AIDS Behav. 2016;20(1):126–36.

14. Amin A. Addressing gender inequalities to improve the sexual and reproductive health and wellbeing of women living with HIV. Journal of the International AIDS Society. 2015 Dec;18:20302.

15. Orza L, Bewley S, Logie CH, Crone ET, Moroz S, Strachan S, Vazquez M, Welbourn A. How does living with HIV impact on women's mental health? Voices from a global survey. Journal of the International AIDS Society. 2015 Dec;18:20289.

16. Mufson L, Morrison C, Shea E, Kluisza L, Robbins R, Chen Y, et al. Screening for depression with the PHQ-9 in young adults affected by HIV. J Affect Disord. 2022;297(646):276–82.

17. Lofgren SM, Bond DJ, Nakasujja N, Boulware DR. Burden of Depression in Outpatient HIV-Infected adults in Sub-Saharan Africa; Systematic Review and Meta-analysis. AIDS Behav [Internet]. 2020;24(6):1752–64. Available from: https://doi.org/10.1007/s10461-019-02706-2

18. Lofgren SM, Nakasujja N, Boulware DR. Systematic review of interventions for depression for people living with HIV in Africa. AIDS and Behavior. 2018 Jan;22:1-8.

19. Page MJ, McKenzie JE, Bossuyt PM, Boutron I, Hoffmann TC, Mulrow CD, Shamseer L,

Tetzlaff JM, Akl EA, Brennan SE, Chou R. The PRISMA 2020 statement: an updated guideline for reporting systematic reviews. International journal of surgery. 2021 Apr 1;88:105906.

20. Roberts KJ, Smith C, Cluver L, Toska E, Sherr L. Understanding Mental Health in the Context of Adolescent Pregnancy and HIV in Sub-Saharan Africa: A Systematic Review Identifying a Critical Evidence Gap. AIDS Behav [Internet]. 2021;25(7):2094–107. Available from: https://doi.org/10.1007/s10461-020-03138-z

21. Sowa, N. A., Cholera, R., Pence, B. W., & Gaynes BN. Perinatal depression in HIV-infected African women: a systematic review. J Clin Psychiatry. 2015;76(10):1385–96.

22. Adjorlolo S, Setordzi M. Psychosis in adolescents in Africa: A scoping review for current understanding and future directions. Cogent Psychol [Internet]. 2021;8(1). Available from: https://doi.org/10.1080/23311908.2021.1949173

23. Joanna Briggs Institute. Checklist for critical appraisal of tools and research synthesis [Internet]. 2017 [cited 2023 May 4]. Available from: https://jbi.global/critical-appraisal tools

24. Munn Z, MClinSc SM, Lisy K, Riitano D, Tufanaru C. Methodological guidance for systematic reviews of observational epidemiological studies reporting prevalence and cumulative incidence data. Int J Evid Based Healthc. 2015;13(3):147–53.

25. Moola S, Munn Z, Tufanaru C, Aromataris E, Sears K, Sfetic R, et al. Chapter 7: Systematic reviews of etiology and risk. JBI Rev Man [Internet]. 2020; Available from: https://synthesismanual.jbi.global

26. Lockwood C, Munn Z, Porritt K. Qualitative research synthesis: Methodological guidance for systematic reviewers utilizing meta-aggregation. Int J Evid Based Healthc. 2015;13(3):179–87.

27. Rael CT, Roberts S, Ibitoye M, Gorbach PM, Palanee-Phillips T, Harkoo I, et al. Likely clinical depression and HIV-related decline in antiretroviral therapy untreated women who seroconverted during participation in microbicide trials in sub-Saharan Africa. Int J STD AIDS. 2021;32(7):620–8.

28. Casale M, Wild L, Cluver L, Kuo C. Social support as a protective factor for depression among women caring for children in HIV-endemic South Africa. J Behav Med. 2015;38(1):17–27.

29. Kamen C, Arganbright J, Kienitz E, Weller M, Khaylis A, Shenkman T, et al. HIV-related stigma: Implications for symptoms of anxiety and depression among Malawian women. African J AIDS Res. 2015;14(1):67–73.

30. Yemeke TT, Sikkema KJ, Watt MH, Ciya N, Robertson C, Joska JA. Screening for Traumatic Experiences and Mental Health Distress Among Women in HIV Care in Cape Town, South Africa. J Interpers Violence. 2020;35(21–22):4842–62.

31. Tsai AC, Wolfe WR, Kumbakumba E, Kawuma A, Hunt PW, Martin JN, et al. Prospective Study of the Mental Health Consequences of Sexual Violence Among

Women Living With HIV in Rural Uganda. J Interpers Violence. 2016;31(8):1531–53.

32. Rossouw J, Schwartz S, Rao A, Mcingana M, Young K, Hausler H, et al. Exploring the Association between Depression and Social and Biobehavioral HIV Risk Factors among Female Sex Workers in Nelson Mandela Bay Municipality, South Africa. AIDS Res Hum Retroviruses. 2021;37(9):666–75.

33. Spies G, Seedat S. Depression and resilience in women with HIV and early life stress: Does trauma play a mediating role? A cross-sectional study. BMJ Open. 2014;4(2):1–7.

34. Sudfeld CR, Kaaya S, Gunaratna NS, Mugusi F, Fawzi WW, Aboud S, Smithfawzi MC. Depression at antiretroviral therapy initiation and clinical outcomes among a cohort of Tanzanian women living with HIV. AIDS (London, England). 2017 Jan 1;31(2):263.

35. Abiodun O, Sodeinde K, Imhonopi G, Omotosho A, Amaike C. Social isolation is associated with major depressive disorders among women accessing HIV/AIDS care in Nigeria. AIDS care. 2022 Jun 3;34(6):741-5.

36. Opiyo E, Ongeri L, Rota G, Verdeli H, Neylan T, Meffert S. Collaborative Interpersonal Psychotherapy for HIV-Positive Women in Kenya: A Case Study From the Mental Health, HIV and Domestic Violence (MIND) Study. J Clin Psychol. 2016;72(8):779–83.

37. Familiar I, Murray S, Ruisenor-Escudero H, Sikorskii A, Nakasujja N, Boivin MJ, Opoka R, Bass JK. Socio-demographic correlates of depression and anxiety among female caregivers living with HIV in rural Uganda. AIDS care. 2016 Dec 1;28(12):1541-5.

38. Kako PM, Wendorf AR, Stevens PE, Ngui E, Otto-Salaj LL. Contending with psychological distress in contexts with limited mental health resources: HIV-positive Kenyan women's experiences. Issues in mental health nursing. 2016 Jan 2;37(1):2-9.

39. Burgess R, Campbell C. Contextualising women’s mental distress and coping strategies in the time of AIDS: A rural South African case study. Transcult Psychiatry. 2014;51(6):875–903.

40. Williams PP, Brooke-Sumner C, Joska J, Kruger J, Vanleeuw L, Dada S, et al. Young South African women on antiretroviral therapy perceptions of a psychological counselling program to reduce heavy drinking and depression. Int J Environ Res Public Health. 2020;17(7):1–16.

41. Govender K, Durevall D, Cowden RG, Beckett S, Kharsany ABM, Lewis L, et al. Depression symptoms, HIV testing, linkage to ART, and viral suppression among women in a high HIV burden district in KwaZulu-Natal, South Africa: A cross-sectional household study. J Health Psychol. 2022;27(4):936–45.

42. Spies G, Konkiewitz EC, Seedat S. Incidence and Persistence of Depression Among Women Living with and Without HIV in South Africa: A Longitudinal Study. AIDS Behav. 2018;22(10):3155–65.

43. Ortblad KF, Musoke DK, Chanda MM, Ngabirano T, Velloza J, Haberer JE, et al. Knowledge of HIV Status Is Associated with a Decrease in the Severity of Depressive Symptoms among Female Sex Workers in Uganda and Zambia. J Acquir Immune Defic

Syndr. 2020;83(1):37–46.

44. Bhardwaj A, Comins CA, Guddera V, Mcingana M, Young K, Phetlhu R, et al. Correction to: Prevalence of depression, syndemic factors and their impact on viral suppression among female sex workers living with HIV in eThekwini, South Africa (BMC Women's Health, (2023), 23, 1, (232), 10.1186/s12905-023-02392-2). BMC Womens Health. 2023;23(1):1–11.

45. Ambrose PR, Cuca YP, Baguso GN, Hoffmann TJ, Dawson-Rose C. Resilience, physical activity, and depression in women living with HIV in the San Francisco Bay Area: a cross-sectional study. Journal of the Association of Nurses in AIDS Care. 2022 Mar 1;33(2):202-10.

46. Heer E, Kaida A, O'Brien N, Kleiner B, Pierre A, Rouleau D, Burchell AN, Skerritt L, Proulx-Boucher K, Nicholson V, Loutfy M. Prevalence of physical health, mental health, and disability comorbidities among women living with HIV in Canada. Journal of Personalized Medicine. 2022 Aug 6;12(8):1294.

47. Vu H, Shaya FT. Predicting Factors of Depression, Antidepressant Use and Positive Response to Antidepressants in Perinatal and Postpartum Women. Clin Pract Epidemiol Ment Heal. 2017;13(1):49–60.

48. Abas M, Ali GC, Nakimuli-Mpungu E, Chibanda D. Depression in people living with HIV in sub-Saharan Africa: time to act. Trop Med Int Heal. 2014;19(12):1392–6.

49. Jörns-Presentati A, Napp AK, Dessauvagie AS, Stein DJ, Jonker D, Breet E, Charles W, Swart RL, Lahti M, Suliman S, Jansen R. The prevalence of mental health problems in sub-Saharan adolescents: A systematic review. Plos one. 2021 May 14;16(5):e0251689.

50. García-Moreno C, Pallitto C, Devries K, Stöckl H, Watts C, Abrahams N. Global and regional estimates of violence against women: Prevalence and health effects of intimate partner violence and non-partner sexual violence. World Health Organization. [Internet]. 2013. Available from: who.int/iris/bitstream/handle/10665/85239/?sequence=1

51. UNAIDS. Evidence for eliminating HIV-related stigma and discrimination: Guidance for countries to implement effective programmes to eliminate HIV-related stigma and discrimination in six settings. 2020.

52. LeGrand S, Reif S, Sullivan K, Murray K, Barlow ML, Whetten K. A Review of Recent Literature on Trauma Among Individuals Living with HIV. Curr HIV/AIDS Rep. 2015;12(4):397–405.

53. Brown MJ, Serovich JM, Kimberly JA, Hu J. Psychological reactance and HIV-related stigma among women living with HIV. AIDS Care - Psychol Socio-Medical Asp AIDS/HIV. 2016;28(6):745–9.

54. Sweeney SM, Vanable PA. The Association of HIV-Related Stigma to HIV Medication Adherence: A Systematic Review and Synthesis of the Literature. AIDS Behav. 2016;20(1):29–50.

55. Turan B, Budhwani H, Yigit I, Ofotokun I, Konkle-Parker DJ, Cohen MH, et al.

Resilience and Optimism as Moderators of the Negative Effects of Stigma on Women Living with HIV. AIDS Patient Care STDS. 2022;36(12):474–82.

56. Ahinkorah BO, Dickson KS, Seidu AA. Women decision-making capacity and intimate partner violence among women in sub-Saharan Africa. Arch Public Heal. 2018;76(1):1–10.

57. Hatcher AM, Smout EM, Turan JM, Christofides N, Stoeckl H. Intimate partner violence and engagement in HIV care and treatment among women: a systematic review and meta-analysis. Aids. 2015 Oct 23;29(16):2183-94.

58. Bernard C, Dabis F, De Rekeneire N. Prevalence and factors associated with depression in people living with HIV in sub-Saharan Africa: A systematic review and meta-analysis. PLoS One. 2017;12(8):1–22.

59. Martinez P, Tsai AC, Muzoora C, Kembabazi A, Weiser SD, Huang Y, et al. Reversal of the kynurenine pathway of tryptophan catabolism may improve depression in ART-treated HIV-infected ugandans. J Acquir Immune Defic Syndr. 2014;65(4):456–62.

Click here to access/download
Supporting Information
TABLE 6.pdf

Click here to access/download
Supporting Information
TABLE 7.pdf

Click here to access/download
Supporting Information
TABLE 8.pdf

Click here to access/download
Supporting Information
Summary of data extracted from included studies.pdf

Click here to access/download
Supporting Information
Search Terms in the databases.pdf

Click here to access/download
Supporting Information
PRISMA_2020_checklist.pdf
