# Supplementary material for: Mental health burden among females living with HIV and AIDS in sub-Saharan Africa: A systematic review
Source: PLOS Glob Public Health. 2024 Feb 1;4(2):e0002767. doi: 10.1371/journal.pgph.0002767 (PMC10833589; doi:10.1371/journal.pgph.0002767)
Supplement: Attachment — Submitted filename: RESPONSE TO REVIEWERS_PGPH_SA edit.docx [file pgph.0002767.s004.docx]

**RESPONSE TO EDITORS**

We noticed you have some minor occurrence of overlapping text with the following previous publication(s), which needs to be addressed:

- <https://doi.org/10.1080/23311908.2022.2111849>

In your revision ensure you cite all your sources (including your own works), and quote or rephrase any duplicated text outside the methods section. Further consideration is dependent on these concerns being addressed.

We conducted similarity checks with the “turn it in” software. Based on the similarity reports, we have rephrased any overlapping text outside the methods sections as suggested by the editor. Information requiring citation has been cited. One additional reference has been cited and added. In the results section text that was not paraphrased has been qualified with quotation marks as suggested.

UNAIDS. Mental Health and HIV. UNAIDS Programme Coordinating Board. Geneva, Switzerland. 2020 [Internet]. Available from: https://www.unaids.org/sites/default/files/media_asset/Mental_Health_%26_HIV_EN.pdf?cv=1

2. Please provide separate figure files in .tif or .eps format only and remove any figures embedded in your manuscript file. Please also ensure that all files are under our size limit of 10MB. You may leave the figure captions or legends in the manuscript.

For more information about how to convert your figure files please see our guidelines: <https://journals.plos.org/globalpublichealth/s/figures>.

Fig1. The PRISMA flow diagram has been uploaded as a separate file. Size limit not exceeding the limit (10MB)

3. Please ensure that you refer to Table 1 in your text as, if accepted, production will need this reference to link the reader to the table.

Suggestion effected in main manuscript text

4. Please include a copy of Table 5 which you refer to in your text on page 12.

Table 5 was already uploaded as a supplementary file in previous submission labelled as summary of data extracted. We have relabeled it as table 5.

5. Please include your main tables as part of your main manuscript and remove the individual files.

Suggestion effected. Thank you

6. We have noticed that you have uploaded Supporting Information files, but you have not included a list of legends. Please add a full list of legends for your Supporting Information files after the references list.

Thank you. Recommended changes effected

7. Please review your reference list to ensure that it is complete and correct. If you have cited papers that have been retracted, please include the rationale for doing so in the manuscript text, or remove these references and replace them with relevant current references. Any changes to the reference list should be mentioned in the rebuttal letter that accompanies your revised manuscript. If you need to cite a retracted article, indicate the article’s retracted status in the References list and also include a citation and full reference for the retraction notice.

Thank you. We have double checked all our references. We are confident that it is complete and correct. Also, none of the articles cited have been retracted. They are all still available at journal websites, institutional websites and significant databases.

RESPONSE TO REVIEWERS

REVIEWER #1

This manuscript meets PLOS Global Public Health’s publication criteria and has adequate statistical analysis that meets the PLOS Global Public Health’s publication criteria. The manuscript is intelligibly written, but the authors need to address some minor typographical errors identified and some of these are highlighted in yellow in the manuscript.

Thanks for the observation and recommendation. All typographical errors identified have been corrected. We have addressed all the specific comments raised by the reviewer in the main manuscript as well.

Although it was not stated clearly in the article, the authors seemed to use prevalence of depressions and anxiety to indicate the burden of mental health.

Thank you. Anxiety and depression were clearly stated in the introduction to represent the burden of mental health this review focused on. Indeed, the prevalence of mental health issues is an indicator of the burden of mental health, such that the higher the prevalence, the more the burden. This partly explains why low- and middle-income countries have a high burden of mental health.

Although this is acceptable, using other measures such as Quality-Adjusted Life-Years (QALY) and Disability-Adjusted Life-Years (DALY) will have been a strong measure of burden. Did the authors come across articles with QALY and/or DALY measurement and did they try to include such articles?

Thank you. Unfortunately, none of the articles included in this review used the Quality-Adjusted Life-Years (QALY) and Disability-Adjusted Life-Years (DALY).

It would have been good if they had gone a step further to look for and use articles with QALY and DALY measurement and include them.

Thank you for your concern. Articles selected for this review were guided by the predefined search terms and strictly by the inclusion and exclusion criteria. We believe that if there were articles published within 2013-2023 on depression and anxiety that used any of these scales, it would have been captured. From our experience and working knowledge in this area, researchers barely use QALY and DALY in assessing mental health burden. This is particularly true when you consider the literature from low- and middle-income countries.

Results: The presentation of the results should conform to the chronology of the objectives.

Thank you. We have rather rearranged the objectives to conform with the presentation of the results and discussion.

Some statements appear contradictory and need to be looked at again; e.g. on page 14, two prevalence figures are reported for Tanzania different from the 61% reported from Tanzania.

Thank you. This was a typographical error and has been corrected. It should be Uganda and not Tanzania

it was not clear if they were still referring to depression or something else.

Thank you. We are unable to tell what the reviewer means by this statement. It is clear from the subheading that, we were reporting on the prevalence of depression

Again, on the same page 5.7% was reported for Nigeria when 5.9% prevalence rate was reported in the earlier sentence.

Thank you. The different reporting figures were a typographical error. We have corrected it to 5.9%.

On page 16, the percentage of 7 out of 19 need to be reported accurately; it should be 36.6 and not 38.9%.

Thank you for your observation. We have corrected this. 7 out of 19 is 36.8%

Reporting on findings on protective factors needs to be clearer; e.g., while the sentence was almost a direct sentence from the abstract, it is difficult for someone reading it for the first time to identify the four coping mechanisms used by the research participants. It would have been better to write the four coping mechanisms clearer for any reader, and this can be found on page 13-19 of the publication source, specifically on page 13. The authors need to avoid plagiarism and ensure sentences are represented accurately.

Thank you. We have rephrased this statement to reflect the understanding we were trying to communicate in the manuscript. We have also used quotation marks for the four coping mechanisms reported by the original source.

Again, on page 17, there was a problem with the representation of the report from the article. The study referenced was conducted in Tanzania and not Uganda and Zambia as mentioned.

Thank you for your observation. This was an obvious typographical error. We have corrected by reporting the exact country, Tanzania.

Secondly, while the key findings of mortality and severe anaemia were measured with statistical significance in the study, these authors did not mention these in their review. They, however, mentioned the one on oral thrush, which was not statistically significant.

We have amended this portion to reflect the correct representation of findings in the original article and quoted the P-Values to confirm their statistical significance.

The key finding from this study has been highlighted in the manuscript but this is not correct as found in the article. The key finding of this study was “Under the assumption of causality, we estimate 36.1% (95% CI: 13.6-55.1%) of deaths among the study cohort were attributable to depression and its consequences. Depression was not significantly associated with the trajectory of CD4 T-cell reconstitution or the risk of immunologic failure (p-values >0.05).” I recommend this part of the manuscript be rewritten and include the key finding from the article.

Thank you for your concern. We are confident that we have not reported any other finding except what is reported in the original article by the authors. We do not see how the findings quoted here by the reviewer differ from what is reported in the manuscript. The findings below (copied directly from the original article’s abstract) confirm our earlier reports.

“After multivariate adjustment, including social support and stigma, depression at ART initiation was associated with increased risk of mortality [hazard ratio (HR): 1.92; 95% confidence interval (CI): 1.15-3.20; P = 0.01] and incidence of severe anemia (hemoglobin <8.5 g/dl; HR: 1.59; 95% CI: 1.07-2.37; P = 0.02)”.

In addition, the name of the study should be mentioned as it indicates to a large extend to the reader what the study is about.

Thank you. We have identified each article by the in-text citation in accordance with the Vancouver style of referencing. We are not sure of what the reviewer is referring to as the name of the study. However, if the name of the study is the research titles/topics of all the 19 articles, then be assured that the full reference list of each article has been written under the subheading ‘References’ to help readers know what each study is about.

The discussion section was well-written, but need to be arranged according to the objectives of the review.

Thank you. The specific objectives have been arranged to conform to the arrangement in the discussion

Conclusions/recommendations section was well-written. Will the authors consider recommending the development of one common standardised scale/measure for south-Saharan Africa?

Thank you. We have made a recommendation for the development of a single validated tool for Sub-Saharan African regions in the discussion section where this issue was extensively discussed. See page 19

The reviewer did not see Table 5.

Thank you. Table 5 was uploaded as the summary of data extracted in our previous submission. The labeling was the problem. We have labeled it as Table 5 and embedded it in the manuscript as suggested by the editors.

The attached manuscript has other comments for the attention of the authors.

Thank you. We have also addressed all the issues raised in the main manuscript.

Overall, we want to thank the reviewer for making time out of his/her busy schedule to review this paper contributing to the reporting quality of the manuscript. Your efforts and expert advice are greatly appreciated.

REVIEWER #2

This work provides a comprehensive review into the mental health challenges faced by females living with HIV/AIDS in sub-Saharan Africa. The study adopts a rigorous systematic review methodology, adhering to the PRISMA guidelines.
The paper opens with a well-structured introduction that clearly establishes the context and significance of the research topic. The description of the research methods is thorough and supported by pertinent information such as search terms and the PRISMA checklist. The review process's transparency is commendable, particularly in the mention of a third reviewer who resolved discrepancies. The study's consideration of quality appraisal scores for each included study enhances its rigor.

Thank you for your keen observations and recognition of our efforts.

However, there are areas for improvement.

• Clarification of Grey Literature Inclusion: While the paper indicates the inclusion of grey literature, it would be beneficial to clarify the handling of these records within the review process. It is unclear whether data from sources beyond the five reported databases were used, and the handling of grey literature needs more clarification, particularly in the PRISMA diagram. I suggest updating the PRISMA diagram to reflect the inclusion of grey literature, thus ensuring a transparent reporting of the methodology. (You can use the PRISMA 2020 flow diagram for new systematic reviews which included searches of databases, registers and other sources).

Thank you, we have considered using the new PRISMA diagram to make the screening process for articles selected through additional searches more transparent.

• Implications for Future Interventions and Practices: The paper could be strengthened by elaborating on how the study's findings can inform future interventions and practices related to mental health issues among FLWHA. While the study's scope may not have encompassed specific solutions, there is room to expand upon potential avenues for addressing mental health challenges. Consider providing more explicit guidance on how mental health interventions could be cost-effective, feasible, and culturally tailored.

Thank you. We have provided an additional subheading labeled “Implications for future interventions and practices”. Under this subheading, we have discussed and suggested mental health interventions that are deemed cost-effective, feasible, and culturally tailored. Two additional references were cited to discuss the feasibility, cost-effectiveness, and culturally tailored interventions for FLWHA

Jesse DE, Gaynes BN, Feldhousen EB, Newton ER, Bunch S, Hollon SD. Performance of a culturally tailored cognitive‐behavioral intervention integrated in a public health setting to reduce risk of antepartum depression: A randomized controlled trial. Journal of midwifery & women's health. 2015 Oct;60(5):578-92.

Chibanda D, Weiss HA, Verhey R, Simms V, Munjoma R, Rusakaniko S, Chingono A, Munetsi E, Bere T, Manda E, Abas M. Effect of a primary care–based psychological intervention on symptoms of common mental disorders in Zimbabwe: a randomized clinical trial. Jama. 2016 Dec 27;316(24):2618-26.

• Practical Application: Expanding upon the recommendation for mental health care integration, offer concrete examples or referenced instances where such integration has been successful in similar contexts. This will provide practical insights for mental health service providers and stakeholders working with this population, aligning with the overarching purpose of systematic reviews.

Thank you. We have provided referenced instances from both low and middle-income countries. This is discussed in the last paragraph of the discussion. These additional references were cited to support our discussion on the practical application of mental health service integration in HIV care.

Conteh, N.K., Latona, A. & Mahomed, O. Mapping the effectiveness of integrating mental health in HIV programs: a scoping review. BMC Health Serv Res**,** 2023; **23**, 396. <https://doi.org/10.1186/s12913-023-09359-x>

Andersen LS, Magidson JF, O’Cleirigh C, Remmert JE, Kagee A, Leaver M, et al. A pilot study of a nurse-delivered cognitive behavioral therapy intervention (Ziphamandla) for adherence and depression in HIV in South Africa. J Health Psychol. 2018;23(6):776–87.

Odokonyero R, Wagner G, Ngo V, Nakasujja N, Musisi S, Akena D. Giving “sadness” a name: the need for integrating depression treatment into HIV care in Uganda. J Int Association Providers AIDS Care (JIAPAC). 2015;14(2):108–11.

Overall, this paper is a valuable contribution to the understanding of mental health challenges faced by FLWHA in sub-Saharan Africa. It sheds light on the prevalence of depression and anxiety, identifies key correlates and predictors of mental health outcomes, and emphasises the need for culturally tailored interventions. Given the high prevalence rates of mental health issues and their impact on HIV progression, the integration of mental health care into routine HIV care is a logical and essential step.

Thank you very much once again for your observation

**Recommendation:** I recommend accepting this paper with minor revisions to address the areas for improvement mentioned above. In summary, the authors should provide more clarity on the inclusion of data sources beyond the five databases reported and the handling of grey literature. Additionally, they should further elaborate on how the findings can inform future interventions and practices, especially in terms of cost-effective and culturally sensitive mental health care for this population in sub-Saharan Africa.

Thank you. We have done that under the subheading “Implications for future interventions and practices”.

We wish to acknowledge your expert advice and time spent reviewing this manuscript which contributes to the overall reporting quality of the paper. We greatly appreciate the sacrifice.
